# Hypomethylation of *ABCG1* in peripheral blood as a potential marker for the detection of coronary heart disease

**DOI:** 10.1186/s13148-023-01533-6

**Published:** 2023-07-28

**Authors:** Jialie Jin, Xiaojing Zhao, Chao Zhu, Mengxia Li, Jinxin Wang, Yao Fan, Chunlan Liu, Chong Shen, Rongxi Yang

**Affiliations:** 1 Department of Epidemiology, School of Public Health, Nanjing Medical University, Nanjing, 210000 People’s Republic of China; 2 Military Translational Medicine Lab, Medical Innovation Research Division, Chinese PLA General Hospital, Beijing, 100000 People’s Republic of China; 3 Beijing Key Laboratory of Chronic Heart Failure Precision Medicine, Medical Innovation Research Division, Chinese PLA General Hospital, Beijing, 100000 People’s Republic of China; 4 Department of Cardiology, Beijing Friendship Hospital, Capital Medical University, Beijing, 100000 People’s Republic of China; 5 Department of Cardiology, The Second Medical Centre, Chinese PLA General Hospital, Beijing, 100000 People’s Republic of China; 6 Division of Clinical Epidemiology, Affiliated Geriatric Hospital of Nanjing Medical University, Nanjing, 210000 People’s Republic of China; 7 Department of Epidemiology, Center for Global Health, School of Public Health, Nanjing Medical University, 211166 Nanjing, China

**Keywords:** DNA methylation, *ABCG1* gene, Coronary heart disease, Peripheral blood, Early detection

## Abstract

**Background:**

Novel molecular biomarkers for the risk assessment and early detection of coronary heart disease (CHD) are urgently needed for disease prevention. Altered methylation of ATP-binding cassette subfamily G member 1 (*ABCG1*) has been implicated in CHD but was mostly studied in Caucasians. Exploring the potential relationship between *ABCG1* methylation in blood and CHD among the Chinese population would yield valuable insights.

**Methods:**

Peripheral blood samples were obtained from a case–control study (287 CHD patients vs. 277 controls) and a prospective nested case–control study (171 CHD patients and 197 matched controls). DNA extraction and bisulfite-specific PCR amplification techniques were employed for sample processing. Quantitative assessment of methylation levels was conducted using mass spectrometry. Statistical analyses involved the utilization of logistic regression and nonparametric tests.

**Results:**

We found hypomethylation of *ABCG1* in whole blood was associated with the risk of CHD in both studies, which was enhanced in heart failure (HF) patients, female and younger subjects. When combined with baseline characteristics, altered *ABCG1* methylation showed improved predictive effect for differentiating CHD cases, ischemic cardiomyopathy (ICM) cases, younger than 60 years CHD cases, and female CHD cases from healthy controls (area under the curve (AUC) = 0.68, 0.71, 0.74, and 0.73, respectively).

**Conclusions:**

We demonstrated a robust link between *ABCG1* hypomethylation in whole blood and CHD risk in the Chinese population and provided novel evidence indicating that aberrant *ABCG1* methylation in peripheral blood can serve as an early detection biomarker for CHD patients.

**Supplementary Information:**

The online version contains supplementary material available at 10.1186/s13148-023-01533-6.

## Background

Cardiovascular diseases (CVDs) and more specifically coronary heart disease (CHD) remain the foremost cause of death and disease burden globally [1, 2]. CHD develops when blood flow in coronary arteries is reduced or blocked, resulting from atherosclerosis, a vascular condition characterized by arterial wall inflammation and the accumulation of plaque [3]. Although the pathogenesis of CHD and atherosclerosis is not completely understood, smoking, alcohol consumption, hypertension, diabetes, hyperlipidemia, and family history have been acknowledged in association with the disease. Risk assessment to identify asymptomatic individuals at high risk of developing CHD is crucial in clinical practice, especially in primary prevention, when different types of interventions are applied [4, 5]. However, environmental and genetic factors, even the well-established ones, can only explain a small proportion of the variability in CHD risk [6]. In addition, the early diagnostic efficiency of main risk factors for CVDs is low, as a large number of CVDs events occur in individuals with a comparatively low or moderate 10-year risk [7, 8]. Therefore, it is necessary to identify novel predictive biomarkers to improve the risk estimation of CHD.

DNA methylation is an essential component of epigenetics and can alter the expression of genes without changing their sequences [9]. As a reversible epigenetic modification, DNA methylation has been shown to link the internal genetic landscape and external environmental exposures [10]. This makes DNA methylation a potential biomarker of environment-related and lifestyle-driven diseases, such as hypertension [11], diabetes [12], and atherogenic dyslipidemia [13]. Recently, several studies have reported the association between DNA methylation patterns in blood samples and CHD or CHD-related risk factors, indicating DNA methylation signatures in peripheral blood would be novel biomarkers for CHD [14–18].

ATP-binding cassette subfamily G member 1 (*ABCG1*) gene encodes a protein which mediates cholesterol efflux to the high-density lipoprotein (HDL) [19, 20], as well as oxysterols and phospholipid transport in macrophages [21, 22]. *ABCG1* prevents the accumulation of excessive cholesterol in the human body and thus prevents the build-up of atherosclerosis [6]. Moreover, Pfeiffer et al. [20] have identified a positive association between the methylation level at the *ABCG1* cg27243685 site and total triglyceride (TG) levels in a German population. Later, a comprehensive EWAS carried out in the USA and Europe revealed that increased methylation level of cg27243685 in blood was inversely associated with *ABCG1* expression and further associated with higher risk of incident CHD [23]. Recently, Ochoa-Rosales et al. and Schrader et al. showed DNA methylation at cg27243685 in peripheral blood was associated with statin therapy, which can effectively reduce the risk of CVDs [24, 25]. Since genetic background and lifestyles may affect DNA methylation patterns [26–28], it would be meaningful to assess the association between *ABCG1* methylation in blood cells and CHD in other ethnic group, especially in prospective study designs. The primary objective of this study was to investigate the association between *ABCG1* methylation levels in blood (including cg27243685 and adjacent CpG sites) and CHD. Initially, a case–control study was conducted to explore this association, followed by the validation of the obtained results through a prospective nested case–control study in the Chinese population.

## Results

### Characteristics of the study subjects

Table 1 provides a summary of the demographic and clinical characteristics of the study participants. In the case–control study, compared to the 42.0% hypertension in the controls, the burden of hypertension in the CHD cases was as high as 70.7% (*p* = 1.08 × 10^–11^). Meanwhile, the CHD cases had significantly lower total cholesterol (TC) and low-density lipoprotein cholesterol (LDL-C) than the controls (*p* values < 1.00 × 10^–6^). The gender, the levels of TG and high-density lipoprotein cholesterol (HDL-C) were well matched between the cases and controls, whereas slight differences were observed in age, habit of smoking and alcohol consumption, and the burden of diabetes (Table 1).

**Table 1:** Demographic and clinical characteristics of participants

Characteristics	Case–control study			Prospective nested case–control study		
	Controls (N = 277)	CHD cases (N = 287)	*p* value	Controls (N = 197)	CHD cases (N = 171)	*p* value
Age (years)	57.00 (51.00–64.00)	61.00 (53.00–70.00)	**0.001**	62.67 (54.67–67.00)	62.83 (54.25–69.58)	0.060
Gender (male/female)	185/92	183/104	0.451	80/117	74/97	0.605
Smoking, *n* (%)	93 (34.8)^a^	124 (43.2)	**0.044**	46 (23.4)	36 (21.1)	0.597
Alcohol consumption, *n* (%)	119 (44.6)^a^	102 (35.5)	**0.030**	58 (29.4)	47 (27.6)^d^	0.704
Hypertension, *n* (%)	110 (42.0)^b^	203 (70.7)	**1.08E−11**	104 (52.8)	121 (70.8)	**4.20E−04**
Diabetes, *n* (%)	61 (23.4)^c^	91 (31.7)	**0.029**	14 (7.1)	20 (11.7)	0.126
TC (mmol/L)	4.41 (3.71–5.12)	3.92 (3.28–4.62)	**1.00E−06**	4.88 (4.41–5.55)	4.85 (4.20–5.53)	0.458
TG (mmol/L)	1.44 (1.06–2.21)	1.30 (0.99–1.96)	0.058	1.40 (0.93–1.93)	1.39 (1.02–1.89)	0.607
HDL-C (mmol/L)	1.12 (0.92–1.36)	1.07 (0.90–1.29)	0.102	1.43 (1.20–1.74)	1.41 (1.18–1.74)	0.573
LDL-C (mmol/L)	2.84 (2.18–3.45)	2.33 (1.85–2.93)	**2.32E−07**	2.72 (2.31–3.27)	2.66 (2.19–3.34)	0.513

CHD, coronary heart disease; TC, total cholesterol; TG, triglyceride; HDL-C, high-density lipoprotein cholesterol; LDL-C, low-density lipoprotein cholesterol; IQR, interquartile range

Data are presented as number of individuals (%) or IQR. Significant *p *values are in bold

^a^Data missing for 10 participants

^b^Data missing for 15 participants

^c^Data missing for 16 participants

^d^Data missing for 1 participant

In the prospective nested case–control study, there were no significant disparities observed in age, gender, smoking and alcohol consumption, diabetes, or levels of TC, TG, HDL-C, and LDL-C between CHD cases and controls. The burden of hypertension in the CHD cases was as high as 70.8% compared to the 52.8% hypertension in the controls (*p* = 4.20 × 10^–4^, Table 1).

### Hypomethylation of *ABCG1* is associated with CHD in the case–control study

To investigate the association between *ABCG1* methylation in blood cells and CHD, a specific amplicon harboring the cg27243685 (referred to as ABCG1_CpG_3) along with seven neighboring CpG sites was quantitatively assessed using mass spectrometry. The statistical analysis was conducted using two logistic regression models adjusted for different covariates. Model A was adjusted for age, gender, and batch effect, while Model B included all the clinical features analyzed in Table 1 (age, gender, smoking, alcohol consumption, hypertension, diabetes, TC, TG, HDL-C, and LDL-C) and batch effect.

In the case–control study (287 CHD cases vs. 277 controls), we found a significant association between CHD and decreased methylation levels of ABCG1_CpG_7.10 (OR per −10% methylation (95% CI) = 1.54 (1.14–2.07), *p* = 0.005, FDR-adjusted *p* value = 0.020) and ABCG1_CpG_9 (OR per −10% methylation (95% CI) = 1.19 (1.05–1.36), *p* = 0.009, FDR-adjusted *p* value = 0.024) by logistic regression model B (Table 2). There were no notable associations identified between CHD and any of the other *ABCG1* CpG sites (*p* > 0.05 by logistic regression model B, Table 2).

**Table 2:** Methylation difference of *ABCG1* between CHD cases and controls in the case–control study

CpG sites	Controls (*N* = 277) median (IQR)	CHD cases (*N* = 287) median (IQR)	OR (95%CI)^A^ per −10% methylation	*p* value^A^	OR (95%CI)^B^ per −10% methylation	*p* value^B^	FDR
ABCG1_CpG_1	0.86 (0.84–0.88)	0.86 (0.82–0.89)	1.28 (0.95–1.72)	0.104	1.27 (0.91–1.77)	0.163	0.261
ABCG1_CpG_2	1.00 (0.98–1.00)	1.00 (0.99–1.00)	1.17 (0.82–1.66)	0.390	1.06 (0.72–1.55)	0.782	0.935
ABCG1_CpG_3/cg27243685	1.00 (1.00–1.00)	1.00 (1.00–1.00)	0.94 (0.79–1.12)	0.490	0.99 (0.81–1.21)	0.909	0.935
ABCG1_CpG_4	1.00 (1.00–1.00)	1.00 (1.00–1.00)	1.43 (0.83–2.47)	0.198	1.79 (0.86–3.74)	0.119	0.238
ABCG1_CpG_7.10	0.59 (0.55–0.64)	0.57 (0.52–0.61)	1.71 (1.30–2.24)	**1.11E-04**	1.54 (1.14–2.07)	**0.005**	**0.020**
ABCG1_CpG_8	0.53 (0.44–0.68)	0.51 (0.39–0.66)	1.05 (0.96–1.15)	0.251	1.00 (0.91–1.11)	0.935	0.935
ABCG1_CpG_9	0.42 (0.32–0.50)	0.35 (0.26–0.44)	1.25 (1.11–1.41)	**1.74E−04**	1.19 (1.05–1.36)	**0.009**	**0.024**

OR, odds ratio; CI, confidence interval; *FDR*, false discovery rate

Model A: Logistic regression adjusted for age, gender, and batch effect

Model B: Logistic regression adjusted for age, gender, smoking, alcohol consumption, hypertension, diabetes, TC, TG, HDL-C, LDL-C, and batch effect. Significant *p *values are in bold

Subsequently, we assessed the link between *ABCG1* methylation and the condition of heart failure (HF). Among the 287 CHD patients, 204 had experienced HF. ABCG1_CpG_7.10 and ABCG1_CpG_9 showed lower methylation level in HF CHD cases (for ABCG1_CpG_7.10, OR per −10% methylation (95% CI) = 1.82 (1.31–2.53), *p* = 3.94 × 10^–4^; for ABCG1_CpG_9, OR per −10% methylation (95% CI) = 1.32 (1.14–1.53), *p* = 2.72 × 10^–4^, logistic regression model B, Additional file 1: Table S1). In contrast, none of the eight measurable CpG sites was associated with CHD cases without HF (*p* values > 0.05 for all, Additional file 1: Table S1).

### Associations between *ABCG1* methylation and CHD stratified by age and gender in the case–control study

It is widely recognized that age and gender can impact DNA methylation patterns [29, 30]. Therefore, we conducted stratified regression analyses to investigate the associations between *ABCG1* methylation and CHD.

The subjects were stratified based on a cutoff age of 60 years, which corresponded to the median age of all the CHD cases and controls. Among those under the age of 60, CHD cases exhibited significant lower methylation levels at five CpG sites compared to the control group (ABCG1_CpG_1, ABCG1_CpG_7.10, ABCG1_CpG_8, and ABCG1_CpG_9, ORs per −10% methylation ranging from 1.20 to 2.60, *p* < 0.033 for all by logistic regression model B, Additional file 1: Table S2). Among those aged 60 years or above, only ABCG1_CpG_8 displayed higher methylation in CHD cases compared to controls (OR per −10% methylation (95% CI) = 0.76 (0.64–0.91), *p* = 0.003 by logistic regression model B, Additional file 1: Table S2).

Next, we investigated the association between *ABCG1* methylation and CHD when stratified by gender. Among females, CHD cases exhibited reduced methylation levels at two CpG sites than controls (for ABCG1_CpG_1, OR per −10% methylation (95% CI) = 2.00 (1.05–3.83), *p* = 0.036; for ABCG1_CpG_9, OR per −10% methylation (95% CI) = 1.34 (1.08–1.67), *p* = 0.009, logistic regression model B, Additional file 1: Table S3). In the male group, only a borderline association was observed between ABCG1_CpG_7.10 hypomethylation and CHD (OR per −10% methylation (95% CI) = 1.46 (1.00–2.13), *p* = 0.049 by logistic regression model B, Additional file 1: Table S3).

### Hypomethylation of *ABCG1* is associated with CHD in the prospective nested case–control study

To further explore the association observed in the case–control study, a prospective nested case–control study (171 CHD cases vs. 197 controls) was conducted. Consistent with the observation in the case–control study, four of the eight CpG sites in the *ABCG1* amplicon exhibited significantly reduced methylation level in CHD patients compared to the control group (ABCG1_CpG_2, ABCG1_CpG_3/cg27243685, ABCG1_CpG_4, and ABCG1_CpG_8, ORs per −10% methylation ranging from 1.33 to 2.49, *p* < 0.024, FDR-adjusted *p* value < 0.038 for all by logistic regression model B, Table 3). Only one CpG site (ABCG1_CpG_1) showed increased methylation in CHD cases than in the controls (OR per −10% methylation (95% CI) = 0.45 (0.26–0.79), *p* = 0.006, FDR-adjusted *p* value = 0.019 by logistic regression model B, Table 3).

**Table 3:** Methylation difference of *ABCG1* between CHD cases and controls in the prospective nested case–control study

CpG sites	Controls (*N* = 197) median (IQR)	CHD cases (*N* = 171) median (IQR)	OR (95% CI)^A^ per −10% methylation	*p* value^A^	OR (95% CI)^B^ per −10% methylation	*p* value^B^	*FDR*
ABCG1_CpG_1	0.86 (0.84–0.89)	0.87 (0.85–0.89)	0.49 (0.28–0.84)	**0.010**	0.45 (0.26–0.79)	**0.006**	**0.019**
ABCG1_CpG_2	0.95 (0.91–1.00)	0.94 (0.89–0.97)	1.78 (1.21–2.61)	**0.003**	1.58 (1.06–2.36)	**0.024**	**0.038**
ABCG1_CpG_3/cg27243685	1.00 (0.97–1.00)	0.99 (0.92–1.00)	1.60 (1.17–2.20)	**0.004**	1.56 (1.13–2.16)	**0.007**	**0.019**
ABCG1_CpG_4	0.96 (0.91–1.00)	0.93 (0.91–0.95)	2.73 (1.67–4.46)	**6.30E-05**	2.49 (1.51–4.12)	**3.83E-04**	**0.003**
ABCG1_CpG_7.10	0.56 (0.53–0.59)	0.55 (0.53–0.58)	1.75 (1.03–2.99)	**0.040**	1.62 (0.92–2.83)	0.094	0.107
ABCG1_CpG_8	0.39 (0.33–0.48)	0.37 (0.33–0.43)	1.42 (1.13–1.80)	**0.003**	1.33 (1.05–1.70)	**0.021**	**0.038**
ABCG1_CpG_9	0.39 (0.33–0.46)	0.38 (0.31–0.42)	1.21 (0.96–1.53)	0.113	1.15 (0.90–1.47)	0.255	0.255

Model A: Logistic regression adjusted for age, gender, and batch effect

Model B: Logistic regression adjusted for age, gender, smoking, alcohol consumption, hypertension, diabetes, TC, TG, HDL-C, LDL-C, and batch effect. Significant *p* values are in bold

**Fig. 1:**
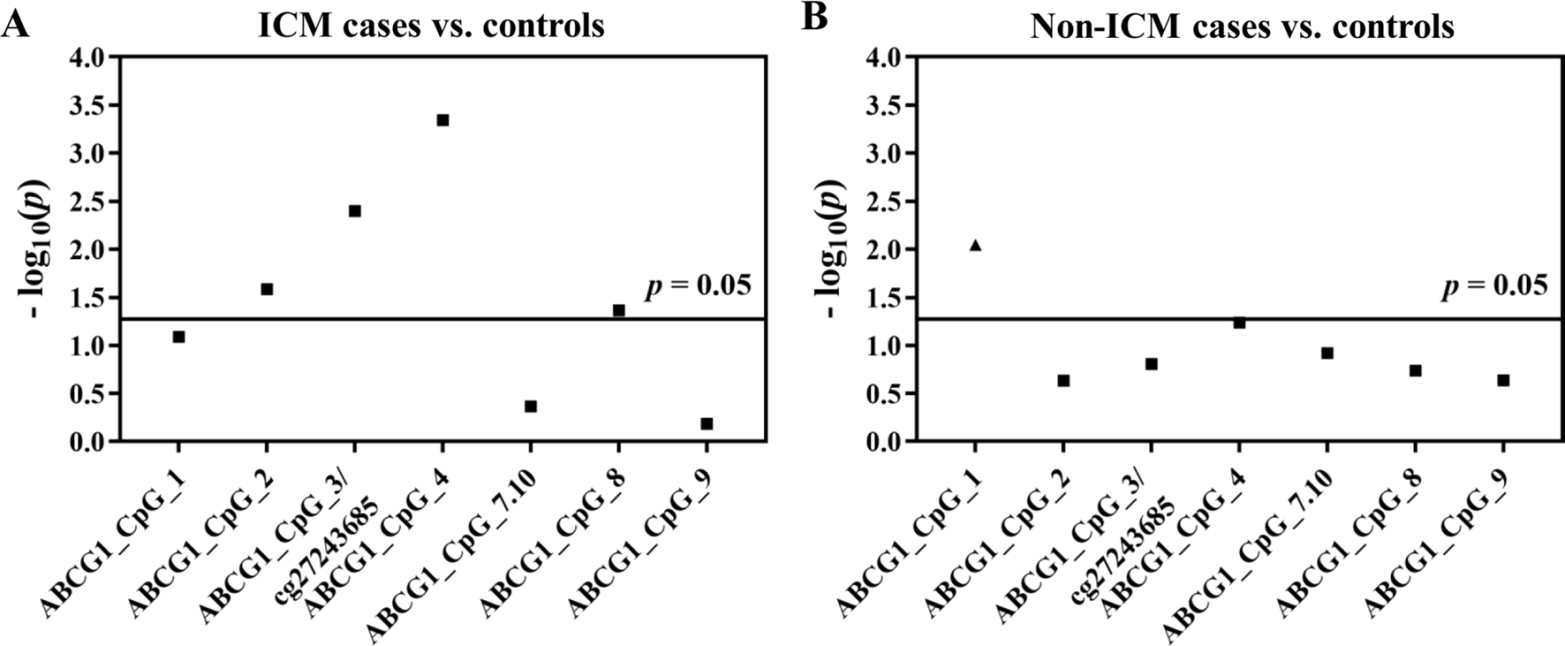
Methylation difference of *ABCG1* between ICM cases (**A**), non-ICM CHD cases (**B**) and controls in the prospective nested case–control study. The point plots showed the methylation differences of CpG sites in the *ABCG1* amplicon. The squares indicated the hypomethylation in cases vs. controls, and the triangles indicated the hypermethylation in cases vs. controls. The *p* values were calculated by logistic regression adjusted for age, gender, smoking, alcohol consumption, hypertension, diabetes, TC, TG, HDL-C, LDL-C, and batch effect. The solid lines indicated the threshold of *p* value = 0.05

Among the 171 CHD patients, 89 were ischemic cardiomyopathy (ICM) cases. The same four CpG sites exhibited decreased methylation level in ICM cases with enhanced ORs (ABCG1_CpG_2, ABCG1_CpG_3/cg27243685, ABCG1_CpG_4, and ABCG1_CpG_8, ORs per −10% methylation ranging from 1.36 to 2.87, *p* < 0.043 for all by logistic regression model B, Fig. 1A, Additional file 1: Table S4). In the non-ICM cases, the methylation level of ABCG1_CpG_1 was increased compared to the controls (OR per −10% methylation (95% CI) = 0.39 (0.19–0.79), *p* = 0.009, by logistic regression model B, Fig. 1B, Additional file 1: Table S4).

### Associations between *ABCG1* methylation and CHD stratified by age and gender in the prospective nested case–control study

**Fig. 2:**
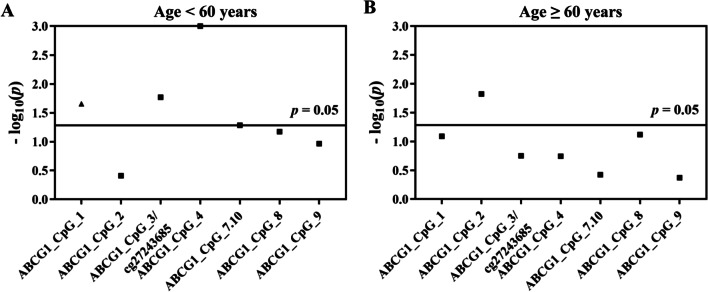
Age-stratified association between *ABCG1* methylation and CHD in the prospective nested case–control study. The point plots showed the methylation differences of CpG sites in the *ABCG1* amplicon. The squares indicated the hypomethylation in cases vs. controls, and the triangles indicated the hypermethylation in cases vs. controls. The *p* values were calculated by logistic regression adjusted for age, gender, smoking, alcohol consumption, hypertension, diabetes, TC, TG, HDL-C, LDL-C, and batch effect. The solid lines indicated the threshold of *p* value = 0.05

We next investigated the association between *ABCG1* methylation and CHD in the follow-up study stratified by age and gender, respectively. In the participants < 60 years old, hypomethylation of ABCG1_CpG_3/cg27243685 and ABCG1_CpG_4 was associated with the risk of CHD (for ABCG1_CpG_3/cg27243685, OR per −10% methylation (95% CI) = 2.07 (1.14–3.77), *p* = 0.017; for ABCG1_CpG_4, OR per −10% methylation (95% CI) = 4.90 (1.89–12.69), *p* = 0.001, logistic regression model B, Fig. 2A, Additional file 1: Table S5). In contrast, ABCG1_CpG_1 showed higher methylation level in CHD cases compared to the control group (OR per −10% methylation (95% CI) = 0.32 (0.12–0.85), *p* = 0.022 by logistic regression model B, Fig. 2A, Additional file 1: Table S5). In the subjects ≥ 60 years old, among the eight CpG sites only ABCG1_CpG_2 showed lower methylation in CHD cases compared to controls (OR per −10% methylation (95% CI) = 2.08 (1.15–3.74), *p* = 0.015 by logistic regression model B, Fig. 2B, Additional file 1: Table S5).

**Fig. 3:**
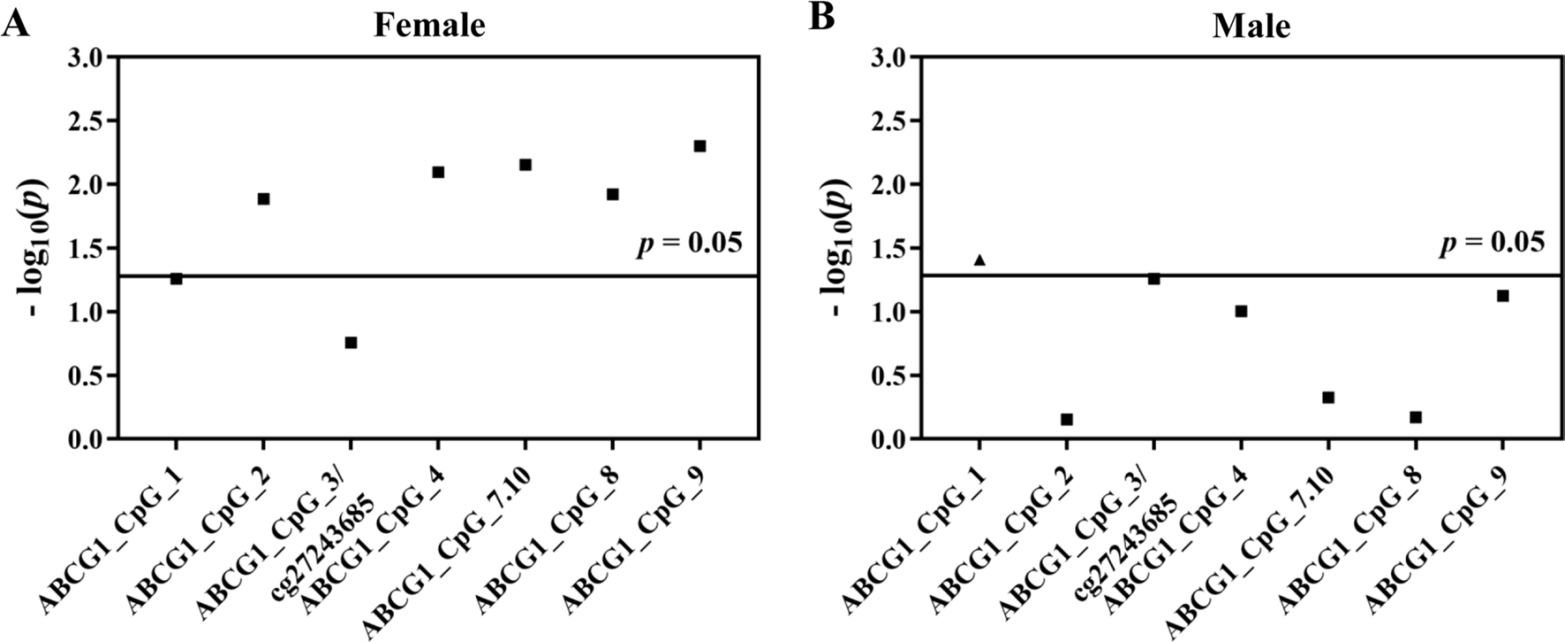
Gender-stratified association between *ABCG1* methylation and CHD in the prospective nested case–control study. The point plots showed the methylation differences of CpG sites in the *ABCG1* amplicon. The squares indicated the hypomethylation in cases vs. controls, and the triangles indicated the hypermethylation in cases vs. controls. The *p* values were calculated by logistic regression adjusted for age, gender, smoking, alcohol consumption, hypertension, diabetes, TC, TG, HDL-C, LDL-C, and batch effect. The solid lines indicated the threshold of *p* value = 0.05

In females, six out of eight CpG loci showed lower methylation in the CHD cases than in the controls (ABCG1_CpG_2, ABCG1_CpG_4, ABCG1_CpG_7.10, ABCG1_CpG_8, and ABCG1_CpG_9, ORs per −10% methylation ranging from 1.58 to 2.93, *p* < 0.013 for all by logistic regression model B, Fig. 3A, Additional file 1: Table S6). In males, hypermethylation of ABCG1_CpG_1 showed the borderline association with CHD (OR per −10% methylation (95% CI) = 0.40 (0.17–0.95), *p* = 0.039, logistic regression model B, Fig. 3B, Additional file 1: Table S6).

### Association between *ABCG1* methylation and CHD stratified by the status of hypertension in the prospective nested case–control study

Given that hypertension is a critical risk factor for CHD and the prevalence of hypertension was distinctly different in incident CHD cases and controls, we further explored the association stratified by hypertension status.

**Fig. 4:**
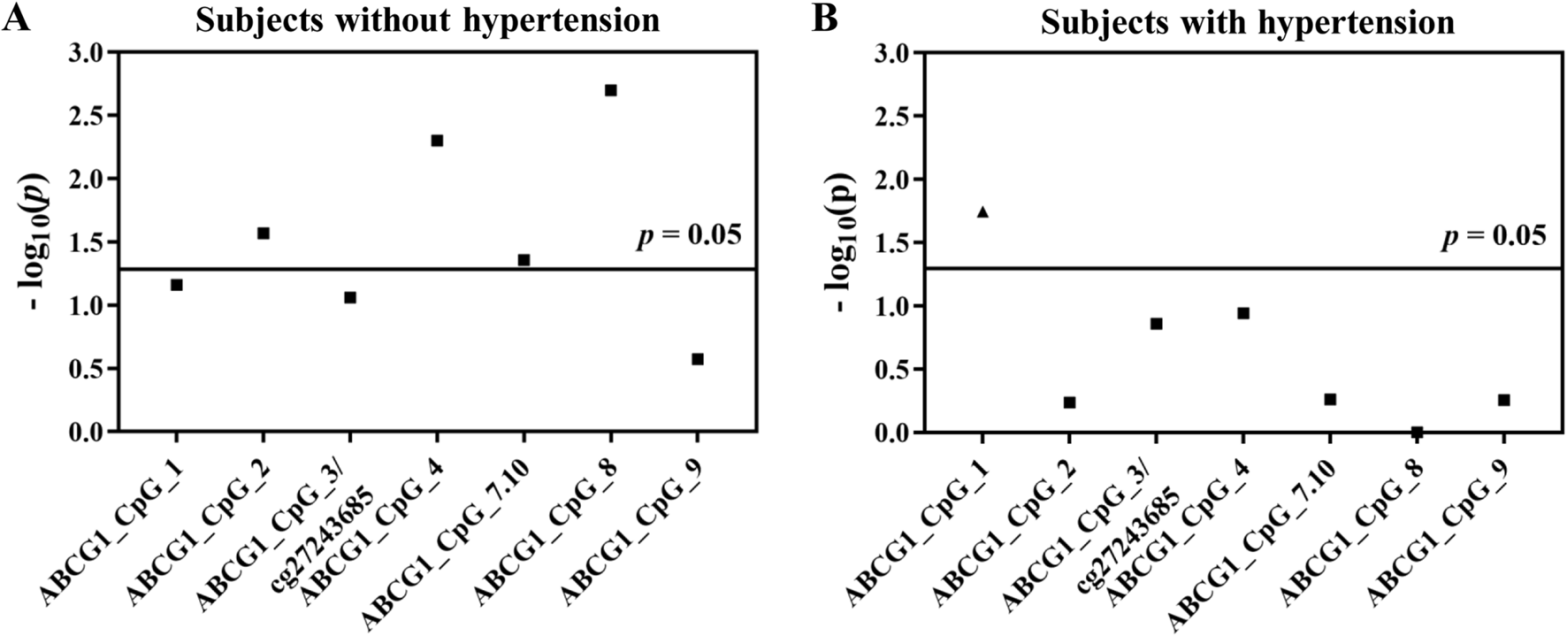
Methylation difference of *ABCG1* between CHD cases and controls in the prospective nested case–control study stratified by the status of hypertension. The point plots showed the methylation differences of CpG sites in the *ABCG1* amplicon. The squares indicated the hypomethylation in cases vs. controls, and the triangles indicated the hypermethylation in cases vs. controls. The *p* values were calculated by logistic regression adjusted for age, gender, smoking, alcohol consumption, hypertension, diabetes, TC, TG, HDL-C, LDL-C, and batch effect. The solid lines indicated the threshold of *p* value = 0.05

Interestingly, hypomethylation of five CpG sites in the *ABCG1* amplicon was significantly associated with CHD in subjects without hypertension (ABCG1_CpG_2, ABCG1_CpG_4, ABCG1_CpG_7.10, and ABCG1_CpG_8, ORs per −10% methylation ranging from 2.02 to 3.44, *p* < 0.044 for all by logistic regression model B, Fig. 4A, Additional file 1: Table S7). In contrast, only hypermethylation of ABCG1_CpG_1 was associated with CHD in the subjects with hypertension (OR per −10% methylation (95% CI) = 0.39 (0.18–0.85), *p* = 0.018 by logistic regression model B, Fig. 4B, Additional file 1: Table S7).

### *ABCG1* methylation as a marker for the detection of CHD in the prospective nested case–control study

**Fig. 5:**
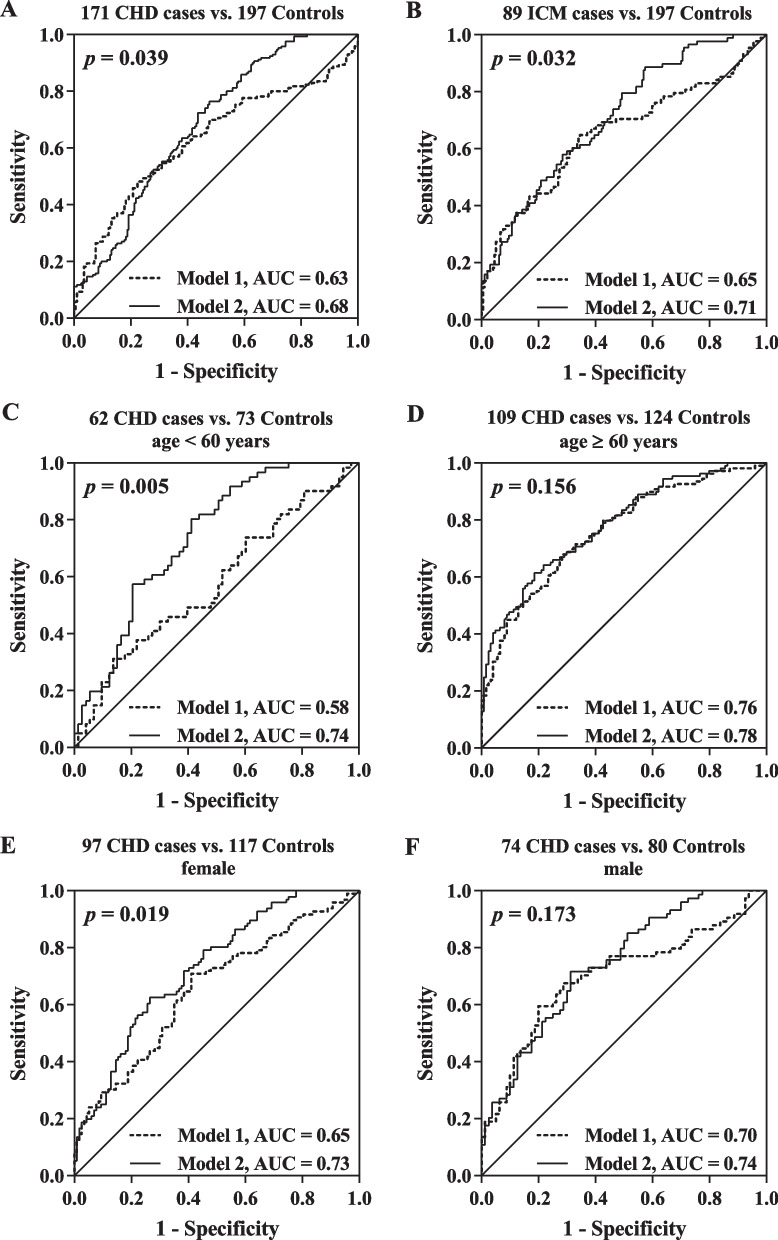
Receiver operating characteristic (ROC) analysis of CHD prediction in the prospective nested case–control study by the model combined *ABCG1* CpG sites and baseline characteristics. The baseline characteristics were assessed as a reference model (Model 1), including age, gender, smoking, alcohol consumption, hypertension, diabetes, TC, TG, HDL-C, and LDL-C. Model 2 combined all *ABCG1* CpG cites with the baseline characteristics. (**A**) Models’ performance for all 171 CHD cases vs. all 197 controls. (**B**) Models’ performance for 89 ICM cases vs. all 197 controls. (**C**) Models’ performance for 62 CHD cases vs. 73 controls in < 60 years old group. (**D**) Models’ performance for 109 CHD cases vs. 124 controls in ≥ 60 years old group. (**E**) Models’ performance for 97 CHD cases vs. 117 controls in females. (**F**) Models’ performance for 74 CHD cases vs. 80 controls in males

In the prospective nested case–control study, we evaluated the potential value of *ABCG1* methylation as an early detection marker for CHD using ROC curve analysis. Two prediction models included in ROC analysis: model 1 with baseline characteristics only (including age, gender, smoking, alcohol consumption, hypertension, diabetes, and the levels of TC, TG, HDL-C, and LDL-C) as the reference model and model 2 with both baseline characteristics and all *ABCG1* CpG cites. Compared to model 1, model 2 showed better predictive effect for differentiating CHD cases, ICM cases, younger than 60 years CHD cases, and female CHD cases from healthy controls (area under curve (AUC) = 0.63, 0.65, 0.58, and 0.65 by prediction model 1, respectively; AUC = 0.68, 0.71, 0.74, and 0.73 by prediction model 2, respectively; *p* = 0.039, 0.032, 0.005, and 0.019, respectively; Fig. 5A–C and E, Additional file 1: Table S8). The discrimination power was similar in two models when analyzed in the subjects older than 60 years old and male group (AUC = 0.76 and 0.70 by prediction model 1, respectively; AUC = 0.78 and 0.74 by prediction model 2, respectively; *p* = 0.156 and 0.173, respectively; Fig. 5D, F, Additional file 1: Table S8).

## Discussion

The present study investigated the potential association between *ABCG1* methylation in blood cells and CHD in a hospital-based case–control study and a prospective nested case–control study. Our findings indicated that hypomethylation of *ABCG1* was associated with the risk of CHD and HF in the Chinese population, particularly in the female and younger subjects.

The ATP-binding cassette protein family encoded by *ABCG1* gene plays an important role in mediating cholesterol transport to the HDL fraction [31]. Hypermethylation of *ABCG1* results in a decrease in gene expression, which is associated with cholesterol metabolism disorders, impaired insulin secretion, and accelerated foam cell formation, and may contribute to type 2 diabetes mellitus, metabolic syndrome, and atherosclerosis CVDs [32–34]. Lower expression of *ABCG1* in human visceral adipose tissue has been shown to be linked to obesity and metabolic syndrome [35]. Kruit et al. [36] found that the HDL-induced increase of insulin secretion relied on *ABCG1*. Loss of both *ABCA1* and *ABCG1* led to the accumulation of sterols, impaired insulin secretion in response to glucose stimulation, and inflammation within the islets. Besides, Miroshnikova et al. [37] have revealed that hypermethylation of *ABCA1* and *ABCG1* in epicardial adipose tissue was associated with coronary artery disease. *ABCA1* and *ABCG1* are both cholesterol transport proteins. *ABCG1* mediates cholesterol transport to the HDL fraction, whereas *ABCA1* regulates the formation of HDL to lipid-free apolipoprotein A-1 [31]. In this study, we have releveled the association between *ABCG1* hypomethylation in blood cells and increased risk of CHD. It would be meaningful to investigate the relationship between *ABCA1* methylation in blood and the risk of CHD in future.

Here, we found decreased methylation level of *ABCG1* was associated with CHD risk in the Chinese population, which was inconsistent with the previously reported CHD-associated *ABCG1* hypermethylation in the American and European population [23, 38]. Zhang et al. [26] have identified notable variations in global genomic DNA methylation among different racial and ethnic groups in peripheral blood. Besides, our previous study has revealed a 22% decrease in the methylation level of S100P_CpG_4 in healthy Chinese individuals compared to Caucasians. Conversely, the methylation level of HYAL2_CpG_2 in Chinese breast cancer cases was found to be approximately 25% higher than that observed in European patients [39]. Collectively, these findings emphasize the importance of validating DNA methylation patterns prior to their application in different ethnic populations. Moreover, Peng et al. [40] have shown that hypermethylation of *ABCG1* promoter is associated with an increased risk of CHD in a Chinese population. Since the amplicon used in our study is located at the first intron of *ABCG1* [37], the differences in the target region may account for the inconsistency of the results.

In the present study, we only observed an association of *ABCG1* hypomethylation with HF CHD cases but not with non-HF CHD cases in the case–control study. Besides, we found that four CpG sites showed decreased methylation in ICM cases with enhanced ORs in the prospective nested case–control study. ICM refers to systolic left ventricular dysfunction in the setting of CHD and represents the most common cause of HF [41]. These results indicate that DNA methylation in blood cells may exert a critical influence on the CHD progression, which needs additional exploration with larger sample size.

Hypertension is a major risk factor for CHD. Interestingly, our results suggested that the association between decreased methylation levels of *ABCG1* and CHD only exist in individuals without hypertension. Xu et al. [33] found the expression of *ABCG1* in peripheral blood monocytes was decreased in the newly diagnosed untreated hypertensive patients. Thus, we proposed that the status of hypertension might be an important confounder for *ABCG1*-associated CHD risk in the Chinese population, and the combination of *ABCG1* methylation and additional risk factors could provide innovative insight into the risk detection of CHD.

Gender differences exist in the prevalence and mortality of CHD [42]. We observed the gender differences in the methylation pattern of *ABCG1* gene between CHD cases and controls. Specifically, the *ABCG1* methylation level in female cases was significantly lower than that in the control group. Jiang et al. [43] revealed that *PLA2G7* methylation was associated with CHD in females, but not in males. Ji et al. [44] identified a significant association between *APOE* hypermethylation and CHD only in the male group. Gender-specific methylation pattern may result from differences in the sex hormones, lifestyle, or environmental factors [45, 46]. Our findings provide further mechanistic support for the gender disparities in incidence and mortality of CHD and suggest the importance of adjusting gender effect when assessing the association between DNA methylation and disease outcomes.

The incidence of CVDs increases in the elder population [2]. However, we observed a significant association between aberrant *ABCG1* methylation and the risk of CHD in individuals younger than 60 years. In our previous study, we revealed the methylation level of ACTB_CpG_4.5 was lower in stroke cases compared to controls in individuals < 65 years, but not in individuals ≥ 65 years [47]. Li et al. [48] also demonstrated the different promoter methylation intensities of *AHCY* in three age groups in peripheral blood. The underlying mechanism of the age-related methylation pattern needs further validation in cohort studies based on substantial sample size.

ROC analysis suggested that the model combined *ABCG1* CpG sites with baseline characteristics showed improved predictive effect for differentiating CHD cases, ICM cases, younger than 60 years CHD cases, and female CHD cases from healthy controls. Our results accorded with the previous study, which identified eight novel CpGs associated with the risk of CVD and having better predictive effect when combined with baseline characteristics in high-risk subjects [49]. Therefore, although *ABCG1* methylation alone may not serve as an adequate screening marker, the combination of additional biomarkers along with CHD-related environmental factors might be helpful for the risk evaluation and early detection of CHD in future. Besides, most CHD patients in the case–control study had a documented medication history (Additional file 1: Table S9). However, we did not find or only find weak influence on methylation by a single drug intake in our study (Additional file 1: Table S9). Own to the limited sample size and the lack of drug-taking time, we cannot investigate whether the combination of different drug intake or the long period of drug taking could have an influence on the methylation. To address these questions, studies with large sample size and follow-up investigations are needed.

A notable strength of this study is that after being identified in a case–control study, the relationship between hypomethylation of *ABCG1* and CHD was further explored in a prospective cohort study. Thus, our findings could provide supportive evidence to chronological order of altered DNA methylation and CHD events. Nevertheless, the fresh blood samples or RNA specimens were not available in this study. The correlation between *ABCG1* methylation and the gene expression in CHD, and the biological function require further investigations. We have noticed that the CHD-associated *ABCG1* CpG sites in the prospective study are variable from the case–control study, which may be attributed to the limited sample size, and may also own to the processing of the CHD since the methylation of *ABCG1* was examined at pre-clinical which is in average 2 years before the events of CHD. Further prospective studies with larger sample sizes are imperative for validation and may also provide additional insights.

## Conclusions

Altogether, our study disclosed the association between *ABCG1* hypomethylation in blood cells and increased risk of CHD in the Chinese population, which was enhanced in HF patients, female and younger subjects. The results provide novel evidence that altered *ABCG1* methylation in peripheral blood might be a potential biomarker for the risk evaluation and early detection of CHD. Further studies based on larger sample size and prospective cohort are needed to validate our findings, and functional experiments are warranted to unravel the underlying molecular mechanisms.

## Methods

### Study populations

Two hundred and eighty-seven CHD patients and 277 gender-matched healthy controls were enrolled in the case–control study (Table1) from Chinese PLA General Hospital during the years of 2018–2019. Since the hospital-based controls were recruited from the health examination which participants are mostly unretired (in China, the age for retirement is 60 years for men and 55 years for women), the ages of the controls were a bit younger than the CHD cases. The median ages of CHD cases, controls, and all the subjects were 61, 57, and 60 years, respectively. The CHD cases were verified through the implementation of coronary angiography in conjunction with clinical manifestations. The medical information for CHD cases is provided in Additional file 1: Table S9. The controls were randomly chosen from annual health examinations whose health reports indicated CHD-free. No additional criteria for inclusion or exclusion were imposed on the control group. Blood samples from all participants were obtained either 8 h after their last meal or following an overnight fast, with the purpose of quantifying the levels of TC, TG, HDL-C, and LDL-C.

Participants for the prospective nested case–control study were collected from a cohort in Jurong City, Jiangsu Province. This particular cohort study was conducted from October to November 2015, and a total of 11,151 individuals aged ≥ 18 years were recruited. Blood samples were obtained at the time point of enrollment when all participants were confirmed to be CHD-free. Demographic information such as age, gender, nationality, smoking status, alcohol consumption, as well as history of hypertension and diabetes, was collected via questionnaires, along with the blood samples at the time of enrollment. Incident CHD was ascertained through coronary angiography conducted by the local hospitals, Centers for Disease Control, and community health service centers. Among the participants enrolled in the cohort, a total of 171 individuals developed CHD within 2.5 years were defined as cases. A total of 197 age- and gender-matched individuals who remained CHD-free according to the health check reports after a median follow-up period of 3.46 years were randomly selected as controls. The median ages of CHD cases and healthy controls were 62.83 and 62.67 years, respectively. Details of this cohort were described in our previous study [47].

### Sample collection and processing

Peripheral blood samples were collected using ethylenediaminetetraacetic acid (EDTA) tubes and preserved at a temperature of -80℃ until DNA isolation. Genomic DNA extraction from peripheral whole blood was performed using DNA Extraction Kit (TANTICA, Nanjing, China). Both case and control samples were handled simultaneously, adhering to the standard manufacturer’s instructions.

### Bisulfite conversion

EZ-96 DNA Methylation Gold Kit (Zymo Research Corporation, Orange, CA, USA) was employed to convert unmethylated cytosine (C) within CpG sites into uracil (U), while methylated cytosine remains intact. The treatment and analysis of both the case and control samples were conducted simultaneously, ensuring parallel processing throughout all stages.

### MALDI-TOF mass spectrometry

Polymerase chain reaction (PCR) was employed to amplify a 356-bp amplicon (chr21: 43642309–43642664, build 37/hg 19, defined by the UCSC Genome Browser) in *ABCG1* gene, which covers cg27243685 along with adjacent CpG sites. The PCR primers for *ABCG1* were designed as follows: forward primer: aggaagagagTGAGTTTAGGAGGTTAAGGAGAAAT, reverse primer: cagtaatacgactcactatagggagaaggctCCAAATCTAAAACCACAAACCTCTA. The specific primer regions were denoted by uppercase letters, while the non-specific tags were indicated in lowercase letters. Notably, no single nucleotide polymorphisms (SNPs) were identified within the primer regions or in overlap with any of the CpG sites. The quantification of methylation levels at *ABCG1* CpG sites was accomplished using Agena MALDI-TOF mass spectrometry (Agena Bioscience, San Diego, CA, USA), following the methodology outlined by Yang et al. [50].

In summary, the PCR products underwent incubation with shrimp alkaline phosphatase (SAP), followed by treatment with the T cleavage assay (Agena Bioscience, San Diego, CA, USA), and subsequent purification using resin. The final products were dispensed onto a 384 SpectroCHIP using a Nanodispenser (Agena Bioscience, San Diego, CA, USA) and subsequently detected by the MassARRAY spectrometry (Agena Bioscience, San Diego, CA, USA). The SpectroACQUIRE v3.3.1.3 software (Agena Bioscience, San Diego, CA, USA) was utilized to obtain the quantitative methylation level of each CpG site, while the EpiTyper v1.2 software (Agena Bioscience, San Diego, CA, USA) was employed for visualization. The MassArray technology produced measurable data for eight CpG sites in *ABCG1* amplicon and yielded seven distinguishable mass peaks. In cases where two CpG sites exhibited identical mass values, we represented their methylation intensities as combined briefly. For instance, ABCG1_CpG_7.10 stands for ABCG1_CpG_7 and ABCG1_CpG_10. The sequence of *ABCG1* amplicon is shown in Additional file 1: Fig. S1. Throughout all processing and analyses, both the CHD cases and controls were managed concurrently. An equivalent number of cases and controls were examined on each chip for the MassARRAY analyses. Furthermore, the present study assessed the reproducibility of methylation analyses conducted via mass spectrometry. For this purpose, a randomly selected DNA sample was measured five times using mass spectrometry, and the standard deviations of all CpG sites were lower than 5% (Additional file 1: Table S10) [51].

### Statistical analysis

Statistical analyses were conducted using SPSS version 25.0 (IBM, NY, USA) and GraphPad Prism 8.0 (GraphPad Software, San Diego, California, USA). Quantitative variables exhibiting a non-Gaussian distribution were presented as median values with interquartile range (IQR). The differences between cases and controls were evaluated using the Mann–Whitney *U* test. The comparison of qualitative variables was carried out using the Chi-square (*χ*^2^) test. Logistic regression analysis was employed to determine the relationship between *ABCG1* methylation and CHD, providing odds ratios (ORs) and 95% confidence intervals (CIs), while also adjusting for covariates. Receiver operating characteristic (ROC) curve analysis was conducted to evaluate the predictive value of altered *ABCG1* methylation levels in CHD outcomes.

## Supplementary Information

**Additional file 1. Table S1:** Methylation difference of *ABCG1* between HF CHD cases, non-HF CHD cases, and controls in the case–control study. **Table S2:** Age-stratified association between *ABCG1* methylation and CHD in the case–control study. **Table S3:** Gender-stratified association between *ABCG1* methylation and CHD in the case–control study. **Table S4:** *ABCG1* methylation in ICM cases and non-ICM cases compared to controls in the prospective nested case–control study. **Table S5:** Age-stratified association between *ABCG1* methylation and CHD in the prospective nested case–control study. **Table S6:** Gender-stratified association between *ABCG1* methylation and CHD in the prospective nested case–control study. **Table S7:** Association between *ABCG1* methylation and CHD stratified by the status of hypertension in the prospective nested case–control study. **Table S8:** The discriminatory power of *ABCG1* methylation to distinguish CHD cases from controls. **Table S9:** The methylation of *ABCG1* in CHD patients with variant medical treatment in the case–control study. **Table S10:** The preparatory experiment of mass spectrometry. **Fig. S1:** Schematic diagram and the sequence of *ABCG1* amplicon.

## Data Availability

The data used and/or analyzed during the current study are available from the corresponding author on reasonable request.

## Abbreviations

AUC: Area under curve
CHD: Coronary heart disease
CIs: Confidence intervals
CVDs: Cardiovascular diseases
HDL-C: High-density lipoprotein cholesterol
HF: Heart failure
ICM: Ischemic cardiomyopathy
IQR: Interquartile range
LDL-C: Low-density lipoprotein cholesterol
MALDI-TOF: Matrix-assisted laser desorption ionization time-of-flight
ORs: Odds ratios
PCR: Polymerase chain reaction
ROC: Receiver operating characteristic
SAP: Shrimp alkaline phosphatase
SNPs: Single nucleotide polymorphisms
TC: Total cholesterol
TG: Triglyceride
