# Supplementary material for: Hypomethylation of ABCG1 in peripheral blood as a potential marker for the detection of coronary heart disease
Source: Clin Epigenetics. 2023 Jul 28;15:120. doi: 10.1186/s13148-023-01533-6 (PMC10375639; doi:10.1186/s13148-023-01533-6)
Supplement: Supplementary file 1 — Additional file 1. Table S1: Methylation difference of ABCG1 between HF CHD cases, non-HF CHD cases, and controls in the case–control study. Table S2: Age-stratified association between ABCG1 methylation and CHD in the case–control study. Table S3: Gender-stratified association between ABCG1 methylation and CHD in the case–control study. Table S4: ABCG1 methylation in ICM cases and non-ICM cases compared to controls in the prospective nested case–control study. Table S5: Age-stratified association between ABCG1 methylation and CHD in the prospective nested case–control study. Table S6: Gender-stratified association between ABCG1 methylation and CHD in the prospective nested case–control study. Table S7: Association between ABCG1 methylation and CHD stratified by the status of hypertension in the prospective nested case–control study. Table S8: The discriminatory power of ABCG1 methylation to distinguish CHD cases from controls. Table S9: The methylation of ABCG1 in CHD patients with variant medical treatment in the case–control study. Table S10: The preparatory experiment of mass spectrometry. Fig. S1: Schematic diagram and the sequence of ABCG1 amplicon. [file 13148_2023_1533_MOESM1_ESM.docx]

**Additional file 1**

**Table S1. Methylation difference of *ABCG1* between HF CHD cases, non-HF CHD cases and controls in the case-control study**

| **CpG sites** | **Controls median (IQR)** | **CHD cases median (IQR)** | **OR (95%CI)^a^ per -10% methylation** | ***p-*value^a^** | **OR (95%CI)^b^ per -10% methylation** | ***p-*value^b^** |
| --- | --- | --- | --- | --- | --- | --- |
| **204 HF CHD cases vs. 277 controls** | | | | | | |
| ABCG1_CpG_1 | 0.86(0.84-0.88) | 0.86(0.82-0.89) | 1.35(0.98-1.85) | 0.069 | 1.33(0.93-1.89) | 0.115 |
| ABCG1_CpG_2 | 1.00(0.98-1.00) | 1.00(0.99-1.00) | 1.16(0.78-1.74) | 0.471 | 1.04(0.68-1.59) | 0.853 |
| ABCG1_CpG_3/cg27243685 | 1.00(1.00-1.00) | 1.00(1.00-1.00) | 0.91(0.71-1.15) | 0.426 | 0.95(0.72-1.25) | 0.705 |
| ABCG1_CpG_4 | 1.00(1.00-1.00) | 1.00(1.00-1.00) | 1.58(0.89-2.80) | 0.115 | 1.94(0.89-4.24) | 0.095 |
| ABCG1_CpG_7.10 | 0.59(0.55-0.64) | 0.56(0.51-0.60) | 2.01(1.48-2.72) | **8.00E-06** | 1.82(1.31-2.53) | **3.94E-04** |
| ABCG1_CpG_8 | 0.53(0.44-0.68) | 0.51(0.39-0.61) | 1.06(0.96-1.18) | 0.248 | 1.02(0.92-1.14) | 0.694 |
| ABCG1_CpG_9 | 0.42(0.32-0.50) | 0.34(0.24-0.42) | 1.38(1.20-1.58) | **5.00E-06** | 1.32(1.14-1.53) | **2.72E-04** |
| **83 Non-HF CHD cases vs. 277 controls** | | | | | | |
| ABCG1_CpG_1 | 0.86(0.84-0.88) | 0.86(0.82-0.89) | 1.22(0.72-2.08) | 0.460 | 0.97(0.51-1.85) | 0.922 |
| ABCG1_CpG_2 | 1.00(0.98-1.00) | 1.00(0.97-1.00) | 1.50(0.78-2.88) | 0.224 | 1.20(0.60-2.39) | 0.602 |
| ABCG1_CpG_3/cg27243685 | 1.00(1.00-1.00) | 1.00(1.00-1.00) | 0.95(0.77-1.16) | 0.591 | 0.97(0.76-1.23) | 0.774 |
| ABCG1_CpG_4 | 1.00(1.00-1.00) | 1.00(1.00-1.00) | 0.96(0.28-3.34) | 0.952 | 1.29(0.31-5.40) | 0.727 |
| ABCG1_CpG_7.10 | 0.59(0.55-0.64) | 0.60(0.56-0.65) | 1.29(0.85-1.96) | 0.236 | 1.03(0.64-1.66) | 0.905 |
| ABCG1_CpG_8 | 0.53(0.44-0.68) | 0.53(0.40-0.75) | 1.09(0.96-1.25) | 0.193 | 1.02(0.87-1.19) | 0.827 |
| ABCG1_CpG_9 | 0.42(0.32-0.50) | 0.38(0.29-0.49) | 1.03(0.86-1.23) | 0.767 | 0.93(0.76-1.13) | 0.453 |
| Abbreviation: HF, heart failure.  Model A: Logistic regression adjusted for age, gender, and batch effect.  Model B: Logistic regression adjusted for age, gender, smoking, alcohol consumption, hypertension, diabetes, TC, TG, HDL-C, LDL-C, and batch effect. Significant *p*-values are in bold. | | | | | | |

**Table S2. Age-stratified association between *ABCG1* methylation and CHD in the case-control study**

| **CpG sites** | **Controls median (IQR)** | **CHD cases median (IQR)** | **OR (95%CI)^a^ per -10% methylation** | ***p-*value^a^** | **OR (95%CI)^b^ per -10% methylation** | ***p-*value^b^** |
| --- | --- | --- | --- | --- | --- | --- |
| **Age < 60 years (129 CHD cases vs. 147 controls)** | | | | | | |
| ABCG1_CpG_1 | 0.86(0.84-0.89) | 0.85(0.81-0.89) | 1.58(1.07-2.34) | **0.022** | 1.66(1.04-2.64) | **0.033** |
| ABCG1_CpG_2 | 1.00(1.00-1.00) | 1.00(1.00-1.00) | 1.44(0.75-2.74) | 0.271 | 1.37(0.60-3.11) | 0.456 |
| ABCG1_CpG_3/cg27243685 | 1.00(1.00-1.00) | 1.00(1.00-1.00) | 0.85(0.69-1.05) | 0.128 | 0.82(0.63-1.06) | 0.131 |
| ABCG1_CpG_4 | 1.00(1.00-1.00) | 1.00(1.00-1.00) | 1.42(0.73-2.73) | 0.299 | 1.67(0.65-4.33) | 0.288 |
| ABCG1_CpG_7.10 | 0.62(0.57-0.66) | 0.60(0.54-0.63) | 2.82(1.84-4.32) | **2.00E-06** | 2.60(1.58-4.28) | **1.71E-04** |
| ABCG1_CpG_8 | 0.65(0.52-0.87) | 0.57(0.41-0.74) | 1.26(1.12-1.42) | **1.26E-04** | 1.20(1.04-1.39) | **0.014** |
| ABCG1_CpG_9 | 0.45(0.36-0.53) | 0.37(0.27-0.47) | 1.42(1.19-1.70) | **1.32E-04** | 1.34(1.08-1.65) | **0.007** |
| **Age ≥ 60 years (158 CHD cases vs. 130 controls)** | | | | | | |
| ABCG1_CpG_1 | 0.86(0.84-0.88) | 0.87(0.83-0.89) | 0.82(0.47-1.42) | 0.470 | 0.79(0.42-1.47) | 0.451 |
| ABCG1_CpG_2 | 1.00(0.96-1.00) | 1.00(0.97-1.00) | 1.13(0.73-1.74) | 0.593 | 1.08(0.67-1.72) | 0.758 |
| ABCG1_CpG_3/cg27243685 | 1.00(1.00-1.00) | 1.00(1.00-1.00) | 2.39(0.82-6.97) | 0.111 | 1.88(0.77-9.69) | 0.532 |
| ABCG1_CpG_4 | 1.00(1.00-1.00) | 1.00(1.00-1.00) | 1.39(0.51-3.77) | 0.522 | 2.52(0.62-10.19) | 0.194 |
| ABCG1_CpG_7.10 | 0.57(0.53-0.60) | 0.55(0.51-0.60) | 1.11(0.74-1.65) | 0.620 | 1.06(0.69-1.64) | 0.779 |
| ABCG1_CpG_8 | 0.45(0.38-0.52) | 0.49(0.39-0.57) | 0.79(0.67-0.93) | **0.005** | 0.76(0.64-0.91) | **0.003** |
| ABCG1_CpG_9 | 0.38(0.26-0.49) | 0.33(0.24-0.43) | 1.13(0.96-1.32) | 0.147 | 1.09(0.92-1.30) | 0.320 |
| Model A: Logistic regression adjusted for age, gender and batch.  Model B: Logistic regression adjusted for age, gender, smoking, alcohol consumption, hypertension, diabetes, TC, TG, HDL-C, LDL-C, and batch effect. Significant *p*-values are in bold. | | | | | | |

**Table S3. Gender-stratified association between *ABCG1* methylation and CHD in the case-control study**

| **CpG sites** | **Controls median (IQR)** | **CHD cases median (IQR)** | **OR (95%CI)^a^ per -10% methylation** | ***p-*value^a^** | **OR (95%CI)^b^ per -10% methylation** | ***p-*value^b^** |
| --- | --- | --- | --- | --- | --- | --- |
| **Female (104 CHD cases vs. 92 controls)** | | | | | | |
| ABCG1_CpG_1 | 0.86(0.84-0.89) | 0.87(0.82-0.89) | 1.72(1.00-2.95) | 0.051 | 2.00(1.05-3.83) | **0.036** |
| ABCG1_CpG_2 | 1.00(0.98-1.00) | 1.00(0.97-1.00) | 1.70(0.82-3.52) | 0.153 | 1.55(0.71-3.38) | 0.267 |
| ABCG1_CpG_3/cg27243685 | 1.00(1.00-1.00) | 1.00(1.00-1.00) | 1.12(0.81-1.54) | 0.497 | 1.15(0.83-1.59) | 0.414 |
| ABCG1_CpG_4 | 1.00(1.00-1.00) | 1.00(1.00-1.00) | 1.26(0.64-2.49) | 0.511 | 1.35(0.49-3.75) | 0.563 |
| ABCG1_CpG_7.10 | 0.60(0.55-0.64) | 0.56(0.51-0.61) | 1.85(1.16-2.96) | **0.010** | 1.69(1.00-2.85) | 0.051 |
| ABCG1_CpG_8 | 0.51(0.41-0.61) | 0.48(0.37-0.58) | 1.04(0.89-1.21) | 0.625 | 1.01(0.85-1.19) | 0.942 |
| ABCG1_CpG_9 | 0.44(0.35-0.53) | 0.34(0.23-0.44) | 1.39(1.14-1.69) | **0.001** | 1.34(1.08-1.67) | **0.009** |
| **Male (183 CHD cases vs. 185 controls)** | | | | | | |
| ABCG1_CpG_1 | 0.86(0.84-0.88) | 0.86(0.83-0.89) | 1.11(0.77-1.61) | 0.573 | 0.97(0.63-1.49) | 0.873 |
| ABCG1_CpG_2 | 1.00(0.98-1.00) | 1.00(0.99-1.00) | 1.01(0.66-1.53) | 0.967 | 0.90(0.57-1.41) | 0.639 |
| ABCG1_CpG_3/cg27243685 | 1.00(1.00-1.00) | 1.00(1.00-1.00) | 0.87(0.70-1.08) | 0.204 | 0.93(0.71-1.22) | 0.607 |
| ABCG1_CpG_4 | 1.00(1.00-1.00) | 1.00(1.00-1.00) | 1.65(0.73-3.69) | 0.226 | 2.01(0.71-5.64) | 0.187 |
| ABCG1_CpG_7.10 | 0.59(0.55-0.63) | 0.58(0.52-0.61) | 1.66(1.19-2.32) | **0.003** | 1.46(1.00-2.13) | **0.049** |
| ABCG1_CpG_8 | 0.54(0.45-0.72) | 0.52(0.41-0.70) | 1.07(0.95-1.19) | 0.267 | 0.99(0.87-1.13) | 0.899 |
| ABCG1_CpG_9 | 0.41(0.31-0.49) | 0.35(0.26-0.44) | 1.18(1.01-1.37) | **0.033** | 1.10(0.93-1.30) | 0.259 |
| Model A: Logistic regression adjusted for age and batch effect.  Model B: Logistic regression adjusted for age, smoking, alcohol consumption, hypertension, diabetes, TC, TG, HDL-C, LDL-C, and batch effect. Significant *p*-values are in bold. | | | | | | |

**Table S4. *ABCG1* methylation in ICM cases and non-ICM cases compared to controls in the prospective nested case-control study**

| **CpG sites** | **Controls median (IQR)** | **Cases median (IQR)** | **OR (95%CI)^a^ per -10% methylation** | ***p-*value^a^** | **OR (95%CI)^b^ per -10% methylation** | ***p-*value^b^** |
| --- | --- | --- | --- | --- | --- | --- |
| **89 ICM cases vs. 197 controls** | | | | | | |
| ABCG1_CpG_1 | 0.86(0.84-0.89) | 0.87(0.85-0.89) | 0.61(0.33-1.14) | 0.121 | 0.56(0.29-1.08) | 0.081 |
| ABCG1_CpG_2 | 0.95(0.91-1.00) | 0.93(0.88-0.97) | 1.89(1.21-2.95) | **0.005** | 1.70(1.07-2.72) | **0.026** |
| ABCG1_CpG_3/cg27243685 | 1.00(0.97-1.00) | 0.98(0.91-1.00) | 1.70(1.21-2.40) | **0.002** | 1.68(1.18-2.39) | **0.004** |
| ABCG1_CpG_4 | 0.96(0.91-1.00) | 0.93(0.90-0.95) | 3.05(1.73-5.36) | **1.08E-04** | 2.87(1.59-5.17) | **4.54E-04** |
| ABCG1_CpG_7.10 | 0.56(0.53-0.59) | 0.55(0.53-0.58) | 1.60(0.84-3.01) | 0.151 | 1.32(0.66-2.63) | 0.431 |
| ABCG1_CpG_8 | 0.39(0.33-0.48) | 0.37(0.32-0.44) | 1.46(1.11-1.93) | **0.007** | 1.36(1.01-1.84) | **0.043** |
| ABCG1_CpG_9 | 0.39(0.33-0.46) | 0.37(0.32-0.42) | 1.17(0.89-1.55) | 0.264 | 1.07(0.79-1.45) | 0.654 |
| **82 Non-ICM cases vs. 197 controls** | | | | | | |
| ABCG1_CpG_1 | 0.86(0.84-0.89) | 0.87(0.85-0.89) | 0.42(0.21-0.83) | **0.013** | 0.39(0.19-0.79) | **0.009** |
| ABCG1_CpG_2 | 0.95(0.91-1.00) | 0.94(0.90-0.97) | 1.61(1.00-2.60) | 0.050 | 1.37(0.82-2.28) | 0.233 |
| ABCG1_CpG_3/cg27243685 | 1.00(0.97-1.00) | 1.00(0.93-1.00) | 1.40(0.93-2.10) | 0.107 | 1.36(0.89-2.07) | 0.156 |
| ABCG1_CpG_4 | 0.96(0.91-1.00) | 0.94(0.91-0.96) | 1.97(1.11-3.50) | **0.021** | 1.80(0.98-3.29) | 0.058 |
| ABCG1_CpG_7.10 | 0.56(0.53-0.59) | 0.55(0.53-0.58) | 1.83(0.94-3.54) | 0.074 | 1.73(0.87-3.45) | 0.120 |
| ABCG1_CpG_8 | 0.39(0.33-0.48) | 0.39(0.34-0.42) | 1.34(1.00-1.79) | 0.052 | 1.24(0.91-1.69) | 0.183 |
| ABCG1_CpG_9 | 0.39(0.33-0.46) | 0.38(0.31-0.42) | 1.24(0.92-1.67) | 0.162 | 1.21(0.89-1.65) | 0.231 |
| Abbreviation: ICM, Ischemic cardiomyopathy.  Model A: Logistic regression adjusted for age, gender, and batch effect.  Model B: Logistic regression adjusted for age, smoking, alcohol consumption, hypertension, diabetes, TC, TG, HDL-C, LDL-C, and batch effect. Significant *p*-values are in bold. | | | | | | |

**Table S5. Age-stratified association between *ABCG1* methylation and CHD in the prospective nested case-control study**

| **CpG sites** | **Controls median (IQR)** | **CHD cases median (IQR)** | **OR (95%CI)^a^ per -10% methylation** | ***p-*value^a^** | **OR (95%CI)^b^ per -10% methylation** | ***p-*value^b^** |
| --- | --- | --- | --- | --- | --- | --- |
| **Age < 60 years (62 CHD cases vs. 73 controls)** | | | | | | |
| ABCG1_CpG_1 | 0.86(0.84-0.88) | 0.88(0.86-0.89) | 0.35(0.14-0.90) | **0.029** | 0.32(0.12-0.85) | **0.022** |
| ABCG1_CpG_2 | 0.96(0.90-1.00) | 0.94(0.90-0.98) | 1.22(0.66-2.25) | 0.525 | 1.32(0.70-2.48) | 0.388 |
| ABCG1_CpG_3/cg27243685 | 1.00(0.97-1.00) | 1.00(0.92-1.00) | 2.00(1.10-3.63) | **0.023** | 2.07(1.14-3.77) | **0.017** |
| ABCG1_CpG_4 | 0.96(0.91-1.00) | 0.93(0.91-0.95) | 4.46(1.83-10.87) | **0.001** | 4.90(1.89-12.69) | **0.001** |
| ABCG1_CpG_7.10 | 0.57(0.53-0.59) | 0.55(0.53-0.57) | 2.13(0.90-5.02) | 0.085 | 2.45(0.99-6.06) | 0.052 |
| ABCG1_CpG_8 | 0.39(0.32-0.48) | 0.37(0.33-0.42) | 1.39(0.93-2.07) | 0.107 | 1.49(0.97-2.29) | 0.067 |
| ABCG1_CpG_9 | 0.39(0.33-0.48) | 0.36(0.31-0.44) | 1.30(0.89-1.89) | 0.179 | 1.38(0.93-2.05) | 0.108 |
| **Age ≥ 60 years (109 CHD cases vs. 124 controls)** | | | | | | |
| ABCG1_CpG_1 | 0.86(0.84-0.89) | 0.87(0.85-0.89) | 0.56(0.27-1.13) | 0.105 | 0.50(0.23-1.09) | 0.081 |
| ABCG1_CpG_2 | 0.95(0.91-1.00) | 0.93(0.88-0.97) | 2.72(1.58-4.69) | **3.28E-04** | 2.08(1.15-3.74) | **0.015** |
| ABCG1_CpG_3/cg27243685 | 1.00(0.96-1.00) | 0.99(0.92-1.00) | 1.45(0.98-2.13) | 0.060 | 1.33(0.88-2.00) | 0.177 |
| ABCG1_CpG_4 | 0.96(0.91-1.00) | 0.94(0.91-0.96) | 1.95(1.07-3.54) | **0.029** | 1.54(0.82-2.88) | 0.180 |
| ABCG1_CpG_7.10 | 0.56(0.53-0.59) | 0.55(0.53-0.58) | 1.66(0.82-3.39) | 0.161 | 1.45(0.64-3.28) | 0.377 |
| ABCG1_CpG_8 | 0.40(0.33-0.48) | 0.38(0.33-0.43) | 1.51(1.12-2.05) | **0.007** | 1.35(0.97-1.89) | 0.076 |
| ABCG1_CpG_9 | 0.39(0.33-0.45) | 0.39(0.33-0.42) | 1.25(0.91-1.72) | 0.161 | 1.16(0.81-1.67) | 0.426 |
| Model A: Logistic regression adjusted for age, gender, and batch effect.  Model B: Logistic regression adjusted for age, smoking, alcohol consumption, hypertension, diabetes, TC, TG, HDL-C, LDL-C, and batch effect. Significant *p*-values are in bold. | | | | | | |

**Table S6. Gender-stratified association between *ABCG1* methylation and CHD in the prospective nested case-control study**

| **CpG sites** | **Controls median (IQR)** | **CHD cases median (IQR)** | **OR (95%CI)^a^ per -10% methylation** | ***p-*value^a^** | **OR (95%CI)^b^ per -10% methylation** | ***p-*value^b^** |
| --- | --- | --- | --- | --- | --- | --- |
| **Female (97 CHD cases vs. 117 controls)** | | | | | | |
| ABCG1_CpG_1 | 0.86(0.84-0.89) | 0.87(0.85-0.89) | 0.56(0.26-1.19) | 0.131 | 0.46(0.20-1.02) | 0.055 |
| ABCG1_CpG_2 | 0.96(0.91-1.00) | 0.94(0.89-0.97) | 2.12(1.27-3.53) | **0.004** | 1.98(1.15-3.40) | **0.013** |
| ABCG1_CpG_3/cg27243685 | 1.00(0.98-1.00) | 0.98(0.93-1.00) | 1.41(0.93-2.13) | 0.103 | 1.34(0.88-2.05) | 0.175 |
| ABCG1_CpG_4 | 0.96(0.91-1.00) | 0.93(0.91-0.95) | 2.93(1.52-5.64) | **0.001** | 2.50(1.27-4.89) | **0.008** |
| ABCG1_CpG_7.10 | 0.57(0.54-0.59) | 0.55(0.53-0.57) | 3.17(1.50-6.73) | **0.003** | 2.93(1.34-6.43) | **0.007** |
| ABCG1_CpG_8 | 0.39(0.33-0.48) | 0.37(0.33-0.41) | 1.62(1.16-2.26) | **0.004** | 1.58(1.11-2.26) | **0.012** |
| ABCG1_CpG_9 | 0.39(0.33-0.47) | 0.36(0.31-0.41) | 1.71(1.22-2.40) | **0.002** | 1.67(1.17-2.39) | **0.005** |
| **Male (74 CHD cases vs. 80 controls)** | | | | | | |
| ABCG1_CpG_1 | 0.85(0.83-0.88) | 0.87(0.85-0.89) | 0.39(0.17-0.89) | **0.025** | 0.40(0.17-0.95) | **0.039** |
| ABCG1_CpG_2 | 0.95(0.90-1.00) | 0.95(0.90-0.97) | 1.35(0.75-2.43) | 0.326 | 1.14(0.59-2.17) | 0.701 |
| ABCG1_CpG_3/cg27243685 | 1.00(0.95-1.00) | 1.00(0.91-1.00) | 1.88(1.12-3.15) | **0.017** | 1.71(0.99-2.94) | 0.055 |
| ABCG1_CpG_4 | 0.96(0.91-1.00) | 0.93(0.91-0.96) | 2.42(1.15-5.11) | **0.020** | 1.99(0.88-4.48) | 0.099 |
| ABCG1_CpG_7.10 | 0.55(0.53-0.59) | 0.55(0.54-0.58) | 0.80(0.36-1.78) | 0.577 | 0.71(0.28-1.80) | 0.474 |
| ABCG1_CpG_8 | 0.40(0.32-0.47) | 0.39(0.33-0.45) | 1.22(0.87-1.70) | 0.255 | 1.08(0.74-1.58) | 0.677 |
| ABCG1_CpG_9 | 0.38(0.32-0.45) | 0.40(0.32-0.45) | 0.80(0.57-1.14) | 0.226 | 0.69(0.46-1.04) | 0.075 |
| Model A: Logistic regression adjusted for age and batch effect.  Model B: Logistic regression adjusted for age, smoking, alcohol consumption, hypertension, diabetes, TC, TG, HDL-C, LDL-C, and batch effect. Significant *p*-values are in bold. | | | | | | |

**Table S7. Association between *ABCG1* methylation and CHD stratified by the status of hypertension in the prospective nested case-control study**

| **CpG sites** | **Controls median (IQR)** | **CHD cases median (IQR)** | **OR (95%CI)^a^ per -10% methylation** | ***p-*value^a^** | **OR (95%CI)^b^ per -10% methylation** | ***p-*value^b^** |
| --- | --- | --- | --- | --- | --- | --- |
| **Subjects without hypertension (50 CHD cases vs. 93 controls)** | | | | | | |
| ABCG1_CpG_1 | 0.86(0.84-0.89) | 0.88(0.85-0.89) | 0.47(0.19-1.17) | 0.105 | 0.41(0.15-1.07) | 0.069 |
| ABCG1_CpG_2 | 0.97(0.94-1.00) | 0.94(0.90-0.97) | 2.38(1.17-4.87) | **0.017** | 2.35(1.10-4.99) | **0.027** |
| ABCG1_CpG_3/cg27243685 | 1.00(1.00-1.00) | 1.00(0.94-1.00) | 1.89(1.05-3.39) | **0.034** | 1.70(0.93-3.13) | 0.087 |
| ABCG1_CpG_4 | 0.99(0.93-1.00) | 0.93(0.91-0.96) | 4.00(1.74-9.23) | **0.001** | 3.44(1.44-8.21) | **0.005** |
| ABCG1_CpG_7.10 | 0.57(0.54-0.59) | 0.55(0.53-0.57) | 3.28(1.19-9.06) | **0.022** | 3.00(1.03-8.73) | **0.044** |
| ABCG1_CpG_8 | 0.44(0.35-0.51) | 0.36(0.32-0.42) | 1.98(1.32-2.98) | **0.001** | 2.02(1.29-3.16) | **0.002** |
| ABCG1_CpG_9 | 0.39(0.34-0.47) | 0.37(0.31-0.43) | 1.34(0.90-1.99) | 0.146 | 1.27(0.83-1.94) | 0.268 |
| **Subjects with hypertension (121 CHD cases vs. 104 controls)** | | | | | | |
| ABCG1_CpG_1 | 0.86(0.84-0.89) | 0.87(0.85-0.89) | 0.44(0.21-0.94) | **0.034** | 0.39(0.18-0.85) | **0.018** |
| ABCG1_CpG_2 | 0.94(0.89-0.97) | 0.94(0.88-0.97) | 1.20(0.73-1.98) | 0.463 | 1.16(0.69-1.93) | 0.579 |
| ABCG1_CpG_3/cg27243685 | 1.00(0.94-1.00) | 0.98(0.92-1.00) | 1.32(0.89-1.96) | 0.171 | 1.36(0.91-2.03) | 0.138 |
| ABCG1_CpG_4 | 0.94(0.91-0.98) | 0.93(0.91-0.95) | 1.61(0.85-3.03) | 0.144 | 1.69(0.88-3.24) | 0.114 |
| ABCG1_CpG_7.10 | 0.55(0.53-0.59) | 0.56(0.53-0.58) | 1.32(0.65-2.66) | 0.441 | 1.27(0.59-2.72) | 0.547 |
| ABCG1_CpG_8 | 0.37(0.31-0.43) | 0.38(0.34-0.43) | 1.03(0.74-1.44) | 0.871 | 1.00(0.70-1.42) | 0.995 |
| ABCG1_CpG_9 | 0.39(0.31-0.44) | 0.38(0.32-0.42) | 1.12(0.82-1.55) | 0.481 | 1.11(0.79-1.57) | 0.554 |
| Model A: Logistic regression adjusted for age, gender, and batch effect.  Model B: Logistic regression adjusted for age, smoking, alcohol consumption, hypertension, diabetes, TC, TG, HDL-C, LDL-C, and batch effect. Significant *p*-values are in bold. | | | | | | |

**Table S8. The discriminatory power of *ABCG1* methylation to distinguish CHD cases from controls**

| **All 171 CHD cases vs. all 197 controls** | |
| --- | --- |
| **Predictive models** | **AUC (95% CI)** |
| Model 1 | 0.63(0.57-0.69) |
| Model 2 | 0.68(0.63-0.74) |
| **89 ICM cases vs. 197 controls** | |
| **Predictive models** | **AUC (95% CI)** |
| Model 1 | 0.65(0.58-0.73) |
| Model 2 | 0.71(0.65-0.77) |
| **62 CHD cases vs. 73 controls, age < 60 years** | |
| **Predictive models** | **AUC (95% CI)** |
| Model 1 | 0.58(0.48-0.67) |
| Model 2 | 0.74(0.65-0.82) |
| **109 CHD cases vs. 124 controls, age ≥ 60 years** | |
| **Predictive models** | **AUC (95% CI)** |
| Model 1 | 0.76(0.70-0.82) |
| Model 2 | 0.78(0.72-0.84) |
| **97 CHD cases vs. 117 controls, female** | |
| **Predictive models** | **AUC (95% CI)** |
| Model 1 | 0.65(0.58-0.73) |
| Model 2 | 0.73(0.66-0.80) |
| **74 CHD cases vs. 80 controls, male** | |
| **Predictive models** | **AUC (95% CI)** |
| Model 1 | 0.70(0.62-0.79) |
| Model 2 | 0.74(0.67-0.82) |
| Model 1: Logistic regression adjusted for all clinical features analyzed in Table 1 (including age, gender, smoking, alcohol consumption, hypertension, diabetes, TC, TG, HDL-C, and LDL-C).  Model 2: Logistic regression adjusted for all clinical features analyzed in Table 1 (including age, gender, smoking, alcohol consumption, hypertension, diabetes, TC, TG, HDL-C, and LDL-C) and all *ABCG1* CpG cites in the amplicon. | |

**Table S9. The methylation of *ABCG1* in CHD patients with variant medical treatment in the case-control study**

| **Medicine** | **Group** | ABCG1_CpG_1 | ABCG1_CpG_2 | ABCG1_CpG_3/cg27243685 | ABCG1_CpG_4 | ABCG1_CpG_7.10 | ABCG1_CpG_8 | ABCG1_CpG_9 |
| --- | --- | --- | --- | --- | --- | --- | --- | --- |
| ACEI | No (N=247) | 0.86 | 1.00 | 1.00 | 1.00 | 0.57 | 0.51 | 0.35 |
|  | Yes (N=40) | 0.87 | 1.00 | 1.00 | 1.00 | 0.55 | 0.49 | 0.35 |
|  | *p*-value | 0.649 | 0.088 | 0.202 | 0.288 | 0.070 | 0.224 | 0.908 |
| ARB | No (N=208) | 0.86 | 1.00 | 1.00 | 1.00 | 0.57 | 0.49 | 0.35 |
|  | Yes (N=79) | 0.87 | 1.00 | 1.00 | 1.00 | 0.59 | 0.54 | 0.35 |
|  | *p*-value | 0.603 | 0.270 | 0.452 | 0.055 | 0.082 | **0.044** | 0.808 |
| CCB | No (N=199) | 0.86 | 1.00 | 1.00 | 1.00 | 0.56 | 0.50 | 0.34 |
|  | Yes (N=88) | 0.86 | 1.00 | 1.00 | 1.00 | 0.58 | 0.52 | 0.37 |
|  | *p*-value | 0.680 | 0.307 | 0.307 | 0.559 | 0.265 | 0.877 | 0.504 |
| β blocker | No (N=105) | 0.86 | 1.00 | 1.00 | 1.00 | 0.58 | 0.51 | 0.36 |
|  | Yes (N=182) | 0.86 | 1.00 | 1.00 | 1.00 | 0.57 | 0.49 | 0.34 |
|  | *p*-value | 0.971 | 0.867 | 0.679 | 0.575 | 0.249 | 0.210 | 0.347 |
| Spironolactone | No (N=235) | 0.86 | 1.00 | 1.00 | 1.00 | 0.57 | 0.51 | 0.35 |
|  | Yes (N=52) | 0.85 | 1.00 | 1.00 | 1.00 | 0.57 | 0.49 | 0.34 |
|  | *p*-value | 0.464 | 0.145 | 0.976 | 0.401 | 0.561 | 0.652 | 0.433 |
| Digoxin | No (N=272) | 0.86 | 1.00 | 1.00 | 1.00 | 0.57 | 0.51 | 0.35 |
|  | Yes (N=15) | 0.86 | 1.00 | 1.00 | 1.00 | 0.58 | 0.48 | 0.26 |
|  | *p*-value | 0.636 | 0.226 | 0.178 | 0.168 | 0.638 | 0.470 | **0.014** |
| Nitrates | No (N=151) | 0.86 | 1.00 | 1.00 | 1.00 | 0.57 | 0.52 | 0.35 |
|  | Yes (N=136) | 0.86 | 1.00 | 1.00 | 1.00 | 0.57 | 0.49 | 0.34 |
|  | *p*-value | 0.640 | 0.404 | **0.004** | 0.306 | 0.865 | 0.205 | 0.967 |
| Aspirin | No (N=64) | 0.87 | 1.00 | 1.00 | 1.00 | 0.56 | 0.52 | 0.32 |
|  | Yes (N=223) | 0.86 | 1.00 | 1.00 | 1.00 | 0.57 | 0.51 | 0.36 |
|  | *p*-value | 0.312 | 0.821 | 0.361 | 0.938 | 0.173 | 0.647 | 0.053 |
| Clopidogrel^#^ | No (N=138) | 0.87 | 1.00 | 1.00 | 1.00 | 0.58 | 0.51 | 0.35 |
|  | Yes (N=148) | 0.86 | 1.00 | 1.00 | 1.00 | 0.57 | 0.51 | 0.35 |
|  | *p*-value | **0.037** | 0.971 | 0.723 | 0.279 | 0.659 | 0.599 | 0.850 |
| Warfarin | No (N=276) | 0.86 | 1.00 | 1.00 | 1.00 | 0.57 | 0.51 | 0.35 |
|  | Yes (N=11) | 0.84 | 1.00 | 1.00 | 1.00 | 0.56 | 0.49 | 0.34 |
|  | *p*-value | 0.309 | 0.575 | 0.410 | 0.934 | 0.309 | 0.560 | 0.235 |
| Statin | No (N=40) | 0.86 | 1.00 | 1.00 | 1.00 | 0.56 | 0.52 | 0.31 |
|  | Yes (N=247) | 0.86 | 1.00 | 1.00 | 1.00 | 0.57 | 0.51 | 0.35 |
|  | *p*-value | 0.719 | 0.491 | 0.840 | 0.499 | 0.421 | 0.611 | 0.134 |
| Antacids | No (N=193) | 0.86 | 1.00 | 1.00 | 1.00 | 0.57 | 0.51 | 0.34 |
|  | Yes (N=94) | 0.86 | 1.00 | 1.00 | 1.00 | 0.57 | 0.51 | 0.36 |
|  | *p*-value | 0.831 | 0.395 | 0.878 | 0.702 | 0.283 | 0.735 | 0.987 |
| Abbreviations: ACEI, Angiotensin converting enzyme inhibitor; ARB, Angiotensin receptor blocker; CCB, Calcium channel blockers.  ^#^ Data missing for 1 participate.  The *p*-values were calculated by Mann-Whitney test, and significant *p*-values are in bold. | | | | | | | | |

**Table S10. The preparatory experiment of mass spectrometry**

| **CpG sites** | ABCG1_CpG_1 | ABCG1_CpG_2 | ABCG1_CpG_3/cg27243685 | ABCG1_CpG_4 | ABCG1_CpG_7.10 | ABCG1_CpG_8 | ABCG1_CpG_9 |
| --- | --- | --- | --- | --- | --- | --- | --- |
| **Methylation level** | 0.90 | 0.98 | 1.00 | 0.99 | 0.57 | 0.36 | 0.29 |
|  | 0.90 | 1.00 | 0.99 | 1.00 | 0.60 | 0.36 | 0.28 |
|  | 0.92 | 0.97 | 0.99 | 1.00 | 0.59 | 0.34 | 0.24 |
|  | 0.87 | 0.98 | 0.96 | 1.00 | 0.59 | 0.35 | 0.29 |
|  | 0.87 | 0.98 | 1.00 | 1.00 | 0.61 | 0.36 | 0.26 |
| **Standard deviation** | 0.022 | 0.011 | 0.016 | 0.004 | 0.015 | 0.009 | 0.022 |
| A randomly selected DNA sample was measured by mass spectrometry for five times. | | | | | | | |

**A**


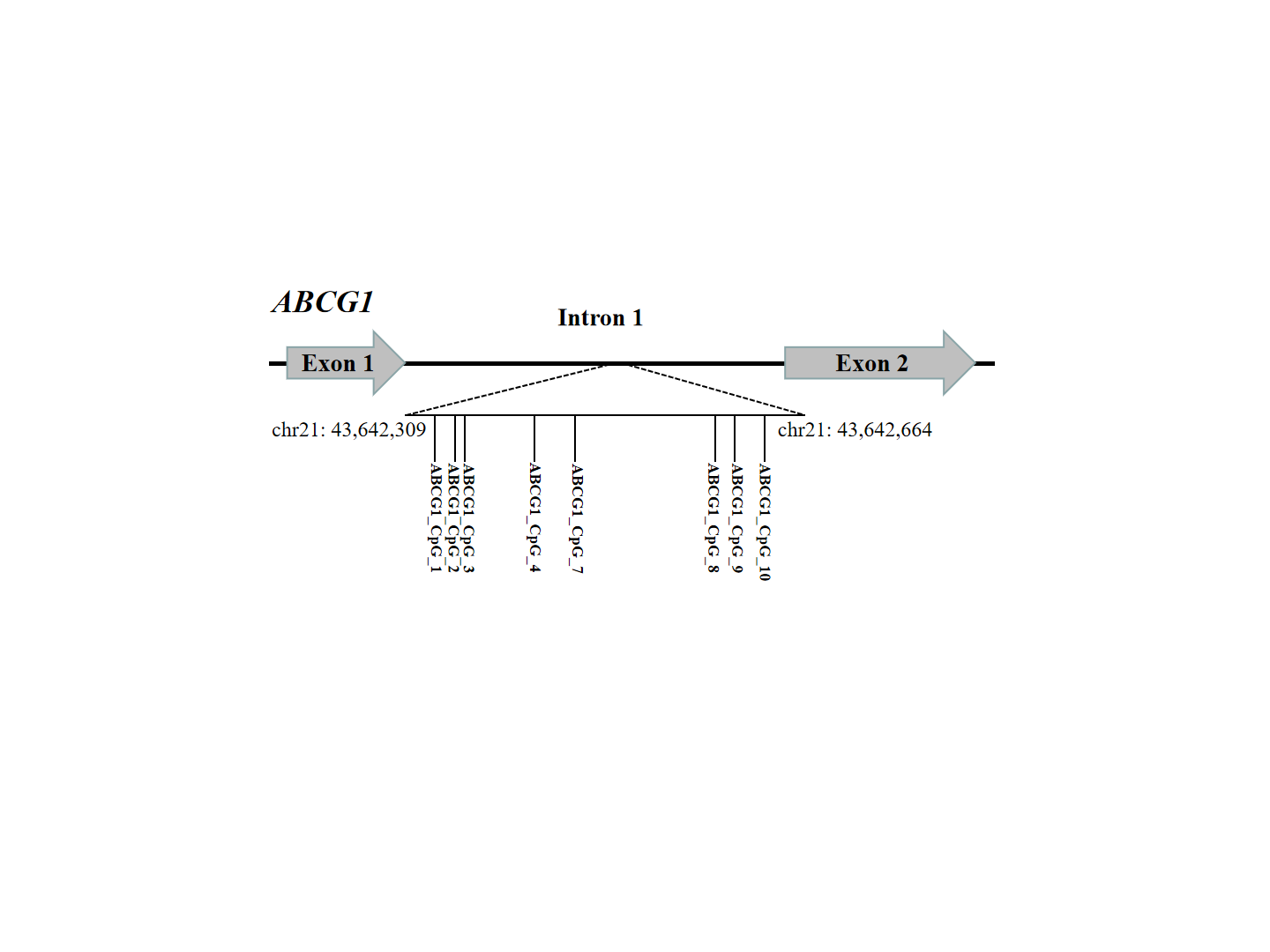


**B**

TGAGCTCAGGAGGCTAAGGAGAAACT**CG**CTGTAACCTACATTGC**CG**GCAGCTCTCAT**CG**GAGGATGTGTTTCCAGAGAGCTGGTCTATTTCAGACTGACAGGCCACTGCCT**CG**CATCACGCAGTTTTCACGATCCTATTAATTGGGTGAGGCTGTTAGA**CG**CTGTGGAGGAAAGGTGGTCAACTGTATGCATTGCTTTGCTGTTGGCTTATTTGGGGATGGGGCTGTATGATTTCCTTGGTGTGGATTTTCCTATAATGGACAAGGGCTGAAAGATGC**CG**AAACACCCACAACTCACC**CG**TGGAAGGCTAAGCAATAGTCAAGGGC**CG**TTACAGAGGCTTGTGGTCTCAGACCTGG

**Fig. S1.** Schematic diagram and the sequence of *ABCG1* amplicon. (A) The location of the investigated 356 bp amplicon in *ABCG1* and the eight measurable CpG sites; (B) The sequence of the *ABCG1* amplicon examined by the EpiTyper assay (chr21: 43642309-43642664, build 37/hg 19, defined by the UCSC Genome Browser). The MassARRAY assay determined the methylation levels of eight CpG sites and yielded seven distinguishable peaks. CpG sites that could be measured are depicted in blue. ABCG1_CpG_3 equals cg27243685.
